# Primary bilateral fronto‐temporoparietal decompressive craniectomy—An alternative treatment for severe traumatic brain injury: Case report and technical note

**DOI:** 10.1002/ccr3.2143

**Published:** 2019-04-16

**Authors:** Rebeca Pérez‐Alfayate, Kita Sallabanda‐Diaz

**Affiliations:** ^1^ Department of Neurological Surgery Hospital Clínico San Carlos Madrid Spain

**Keywords:** craniectomy, decompressive craniectomy, functional outcomes, intracranial hypertension and traumatic brain injury

## Abstract

Bilateral fronto‐temporoparietal decompressive craniectomy provides bigger area of the decompression that decreases the brain tissue herniation; therefore, it leads to a decrease in the neuronal stretching effect that is probably related to functional outcomes.

## 1 INTRODUCTION

Traumatic brain injuries are one of the causes of major disabilities in industrialized countries, creating elevated costs for the population.^1^, ^2^ During the past few decades, there has been an increase in the interest to determine the initial features that could predict surgical outcomes and the optimal treatment to control intracranial pressure, which is a secondary result in response to cerebral edema. The increase in intracranial pressure leads to ischemia by decreasing the cerebral perfusion pressure.^3^, ^4^ Medical and surgical therapies are performed to minimize secondary brain injury.^5^ Elevated intracranial pressure during the acute period is one of the most important predicting factors of mortality and severe morbidity after traumatic brain injury. There is a negative relationship between intracranial pressure and mortality.^1^, ^6^, ^7^, ^8^

Presently, the European Brain Injury Consortium and Brain Trauma Foundation guidelines for severe traumatic brain injuries refer to decompressive craniectomy as a second‐tier therapy for refractory intracranial hypertension that does not respond to conventional therapeutic measures. When intracranial hypertension occurs and is refractory to the first‐tier therapies, a decompressive craniectomy could control the intracranial pressure. 6, 8, 9 Nevertheless, regaining control of the intracranial pressure can transition the patients, who otherwise will die, to a persistent vegetative state. 8, 10 Factors including a preoperative GCS <6, old age, and long decompression time have been reported to be associated with a high risk of persistent vegetative outcomes.

On the other hand, the exact timing of the decompressive craniectomy has not been established^11^, ^12^; the choice of craniectomy technique also remains controversial.

The decompressive effect primarily depends on the size of the part of the skull that is removed. At present, the more widely used techniques are large (more than 12 cm in diameter) unilateral fronto‐temporoparietal decompressive craniectomies for lesions or swelling that are confined to one cerebral hemisphere and bifrontal craniectomies from the floor of the anterior cranial fossa to the coronal suture to the pterion for diffuse swelling.^13^ A fronto‐temporoparietal decompressive craniectomy could provide better decompression of the subtemporal area than a bicoronal craniectomy to prevent transtentorial herniation of the temporal lobe; additionally, a fronto‐temporoparietal decompressive craniectomy allows for more extensive exposure of the parietal lobe. At the same time, a fronto‐temporoparietal decompressive craniectomy could lead to contralateral extracerebral hematoma effusion by relieving the tamponade effect.^14^, ^15^

A bifrontal decompressive craniectomy is most often indicated for diffuse swelling and allows a good space in the anterior fossa; however, it may not achieve the same level of decompression in the medial fossa as a fronto‐temporoparietal decompressive craniectomy. Cooper et al showed in the DECRA study that bifrontal decompressive craniectomies do not improve outcomes versus conservative management for severe diffuse traumatic brain injuries^6^ (Table 1).

**Table 1:** Review of some of the most relevant studies about decompressive craniectomies after traumatic brain Injuries in the last 10 years

Author/year	Sample size	Type of study	Patients age	Decompressive technique	Threshold ICP	Surface area of the craniectomy	Timing for the DC	Timing for the cranioplasty (median)	Good outcome (GOS:4‐5, GOS‐E:5‐8, mRS 0‐4)	Additional details
Chibbaro et al^1^	147	Prospective multicenter	39 yo	BF (18)/ FPT (129)	20 mm Hg	84 cm^2^	Compares <9 h vs >9 h	6 wk	GOS (4,5) = 45%	DC+ early cranioplasty could be effective. Better results at younger ages. Better outcome if DC in the first 9 h
Cooper et al^6^	155	Prospective randomize multicenter	23.7 yo	BF	20 mm Hg 14.4 mm Hg in craniectomy group	‐	38.1 h from injury	10.94 wk	GOS‐E(5‐8) = 30%	Compares DC vs standard of care in inpatients with severe diffuse traumatic brain injuries and increased intracranial pressure that was refractory to first‐tier therapies. Patients in DC group had shorter duration of mechanical ventilation, shorter stay in the ICU, more frequent hydrocephalus, more frequent unfavorable outcomes and worse functional outcomes than the medical group after 6 mo
Hutchinson et al^8^ 2016	408	Multicenter, parallel‐group, superiority, randomized trial	Surgical group 32.3 ± 13.2 yo Medical Group 34.8 ± 13.7 yo	BF/FPT	25 mm Hg	‐	12 h if ICP remains >25 mm Hg despite first‐tier therapies	‐	GOS‐E(5‐8) = 45.4%	Compares DC vs medical treatment in patients with severe traumatic brain injuries and increased refractory intracranial hypertension. DC resulted in lower mortality and higher rates of vegetative states, lower severe disability, and upper severe disability than medical care after 6 mo
Gouello et al^11^ 2014	60	Cohort Retrospective single center study	33 yo	FPT	20 mm Hg	100 cm^3^	Mean of 2.35 d after TBI in first‐tier therapy group, 0.55 d in the early DC group.	‐	GOS (4,5) = 50%	Compares 2 groups: Group 1 underwent surgery after 2,35 days; and group 2 underwent surgery after 0,55 days. DC has better results when performed after first‐tier therapies
Ahmed et al^18^ 2017	942	Retrospective. Data collected from the National Trauma Data Bank (NTDB) (RDS_2007‐RDS_2010)	Early group 38.4 yo Late group: 40.3 yo	BF/FPT	DC has been performed based on neurological evaluation of the patient with or without ICP monitoring.	‐	Early group <4 h after admission. Late group: 4‐24 h after admission.	‐	‐	No differences were seen in mortality between patients operated on within 4 h versus patients operated on between 4 and 24 h of admission.
Zhang et al^19^ 2016	282	Meta‐analysis	30‐50 yo	BF/FPT	25 mm Hg	‐	Early DC <24 h Late DC >24 h	‐	‐	Bilateral pupil abnormality is associated with unfavorable outcomes and increased mortality in the patients who underwent DCs after moderate and severe TBIs. No significant differences between the early and late group in relation to unfavorable outcomes and mortality.
Sauvigny et al^21^ 2018	102	Retrospective single center	Good outcome: 50.7 ± 16. 6 Bad outcome 54.4 ± 19.6	FPT	‐	No significant differences regarding size of trepanation (good outcome: 128.8 ± 27.7 cm^2^ vs bad outcome: 116.8 ± 25.5 cm^2^)	‐	‐	mRS 0‐4 = 48%	ICP < 15 mm Hg during the first 12 h after a DC is associated with lower mortality. Higher probability of favorable outcomes if ICP is between 10‐17 mm Hg after a DC.
Flint et al^23^ 2008	40	Retrospective	43 ± 17 yo	FPT	20 mm Hg	‐	82% of cases <24 h 62% after admission CT scan	‐	GOS 4‐5 = 38%	An increase in contusion volume greater than 20 cc following hemicraniectomy is strongly associated with patient mortality.
Sedney et al^25^ 2014	20	Retrospective	37.9 yo	FPT	‐	124 cm^2^	‐	‐	GOS 4‐5 = 30%	All patients in this study who underwent a craniectomy and had an AP diameter of less than 10 cm died. Results did not demonstrate a significant relationship between craniectomy size and outcome or complication rate.

## 2 METHOD

All procedures performed in this study were in accordance with the ethical standards of the institutional and/or national research committee (Hospital Clínico San Carlos) and with the 1964 Helsinki declaration and its later amendments or comparable ethical standards. Informed consent was obtained from all participants who were included in the study. Informed consent for the procedure was obtained from the families of the patients, as the patients were not able to decide. No funding was received for this research. The outcomes included mortality and neurological outcomes, which were evaluated 6 months following the surgery.

In this article, two cases are presented in which early bilateral fronto‐temporoparietal decompressive craniectomies were performed for two young patients. Both patients presented with clinical and radiological signs of intracranial hypertension and bilateral extracerebral lesions. Only one side of the bilateral lesions indicated the need for an urgent evacuation of the hematoma, but an early bilateral fronto‐temporoparietal decompressive craniectomy was performed in order to avoid evolution of the contralateral mass lesion and to achieve good intracranial pressure control. Both patients presented extended Glasgow Outcome Scale scores of 8, indicating good upper recovery three months after the intervention. Early cranioplasties were performed in both cases to avoid motor trephine syndrome.

### 2.1 Operative technique

Both patients underwent early bilateral fronto‐temporoparietal decompressive craniectomies in the first 24 hours. In both cases, we started by first performing a craniectomy on the side with the hematoma that had the surgical indication. A trauma flap incision was performed while avoiding damage to the superficial temporal artery in order to preserve the optimal perfusion in the flap. We did not remove the temporal muscle. A 12 cm fronto‐temporoparietal craniectomy was performed while ensuring that the squamous portion of the temporal bone was drilled to the temporal lobe base to release compression on the basilar cisterns. The dura mater was opened, and the hematoma was evacuated. An augmentative duraplasty with DuraGen® (Integra LifeSciences Corp.) was performed, as it has been shown to produce good outcomes, and it has been demonstrated to be useful for creating an ingrowth of connective tissue that has similar properties to the dura mater.^17^, ^18^ The dural substitute was placed between the arachnoid membrane and the opened dura mater to avoid cerebrospinal fluid leakage. The scalp was closed with a single layer technique using nonabsorbable sutures. After the first side was done, the same technique was performed on the contralateral side.

### 2.2 Patients

#### 2.2.1 Case 1

**Figure 1:**
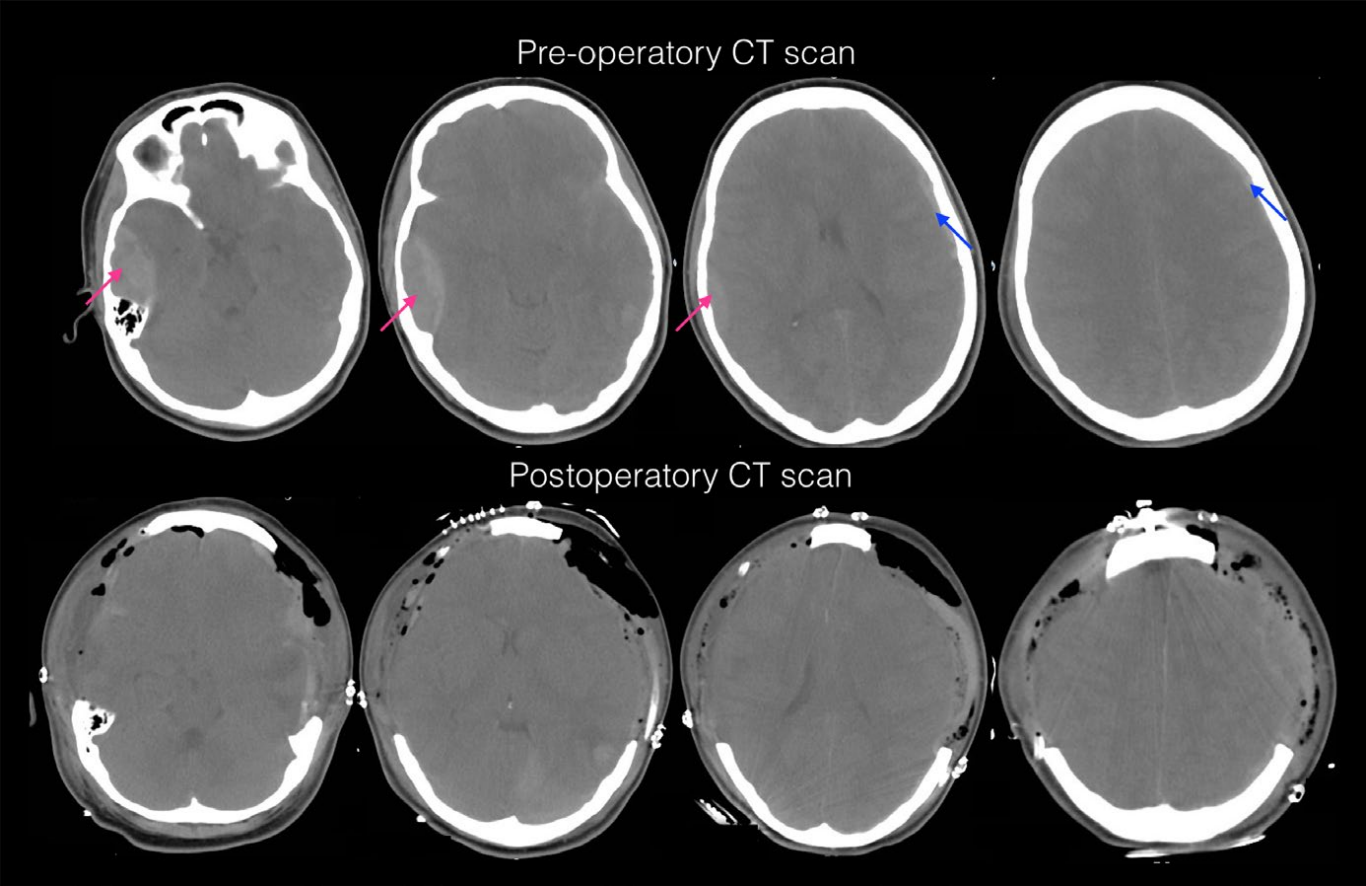
Case 1: Admission CT scan shows a right frontotemporal epidural hematoma that is 2.5 cm in diameter (red arrow), a small left fronto‐parietotemporal subdural hematoma (blue arrow), a left traumatic subarachnoid hemorrhage, and small left brain contusions. Additionally, compressed cisterns can be observed. The CT scan 24 h after surgery. BFTP‐DC was performed. Pneumocephalus and herniation of the brain tissue through the craniectomies can be observed

**Figure 2:**
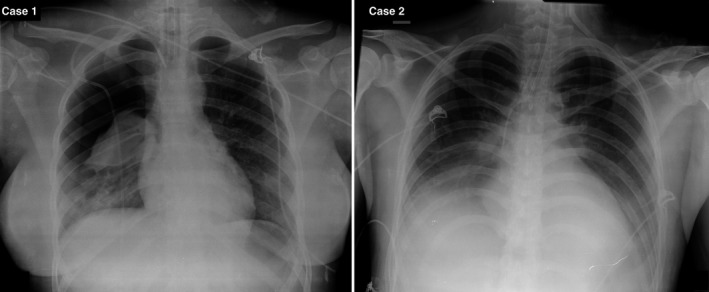
X‐ray case 1: Patient presented a right pneumothorax. X‐ray case 2: Patient presented with pneumonia. Patients kept under sedation after achieving normal ICP because of these findings

A 20‐year‐old woman was run over by a car, lost consciousness, which was regained after a few seconds, and presented with GCS = 12. She was admitted in the emergency room of our institution and presented with a Glasgow Coma Scale score of 9 out of 15. The CT scan showed a Marshall CT classification of diffuse injury IV because of a right frontotemporal epidural hematoma that was 2.5 cm in diameter and a small fronto‐parietotemporal subdural hematoma in addition to a left traumatic subarachnoid hemorrhage, left small brain contusions and fractures of the skull base, right temporal bone and parietal bones (Figure 1). She had a Rotterdam classification score of 3. A ventriculostomy was placed in the ICU while the emergency operating room was being prepared for a neurosurgical procedure and the neurosurgery personnel were arriving at the hospital. Her intracranial pressure was 22 mm Hg. An early bilateral fronto‐temporoparietal decompressive craniectomy was performed, and the extracranial hematomas were evacuated. The patient was kept under sedation and paralysis, she had her cerebrospinal fluid drainage monitored, and she received osmotic diuresis to achieve an intracranial pressure of approximately 10 mm Hg. The patient presented a right pneumothorax (Figure 2), which was resolved with the insertion of a chest tube for drainage. She was extubated after 2 weeks so that her respiratory process and psychomotor dimension could be agitated when the sedation was suppressed for neurological evaluation. After 3 weeks in the intensive care unit, the patient was admitted to the neurosurgical floor, where she received physical therapy. The patient underwent an early cranioplasty repair with an autologous graft 4 weeks after the accident. Six months later, the patient had a score of 8 on the extended Glasgow Outcome Scale, indicating good upper recovery, and a Disability Rating Scale of 0.

#### 2.2.2 Case 2

**Figure 3:**
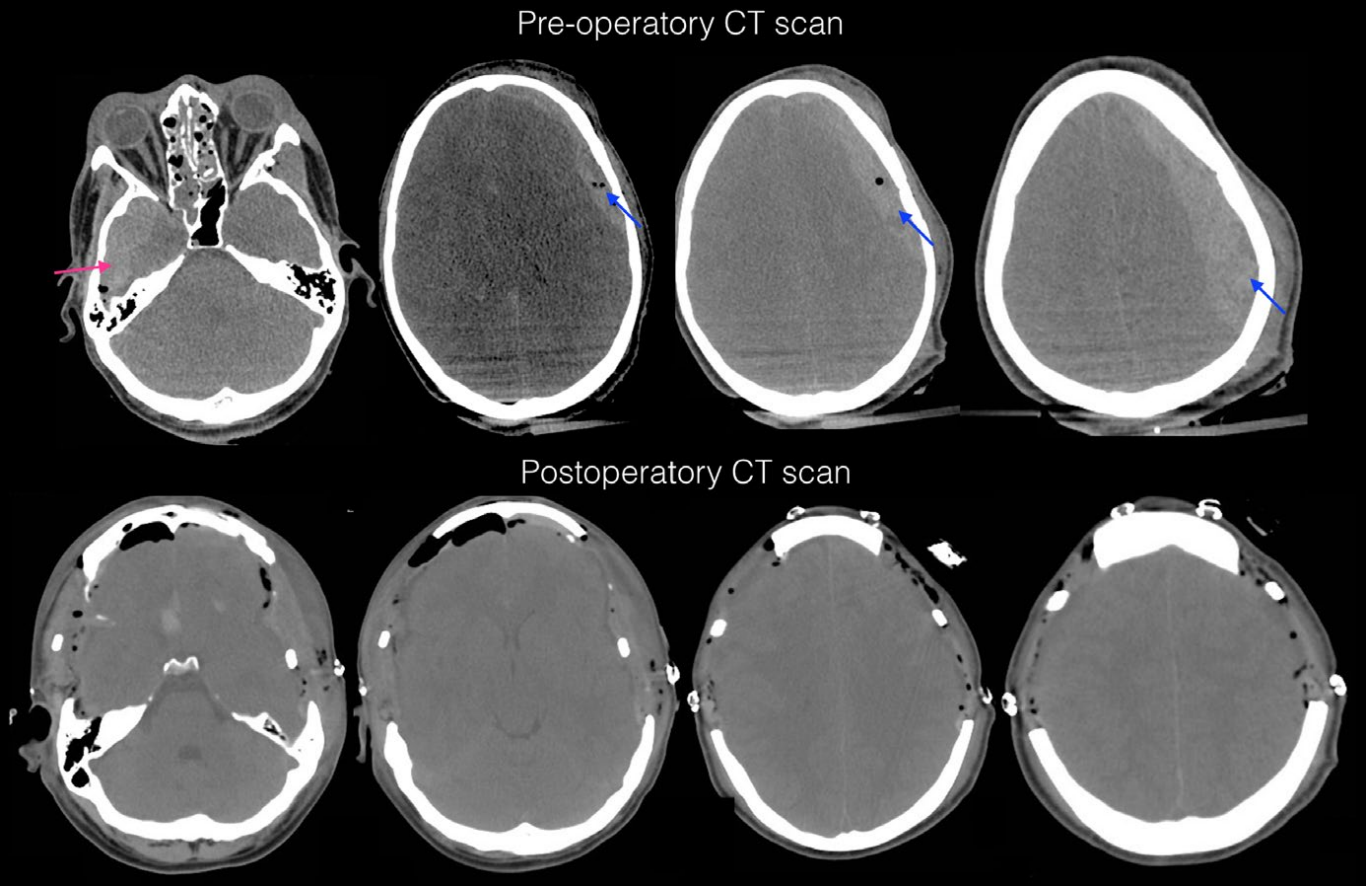
Case 2. Admission CT scan shows a left fronto‐parietotemporal subdural hematoma (blue arrow), a fronto‐basal right contusion, a temporal contusion, a small right temporal epidural hematoma (red arrow), and compressed cisterns. CT scan 24 h after surgery shows a BFTP‐DC. The brain tissue did not herniate through the craniectomies

A 24‐year‐old man was injured in a motorcycle accident in which he did not wear a helmet. He arrived at the emergency room of our institution. He was intubated and presented with a GCS score of 8 out 15 and anisocoria (left mydriasis). The CT scan showed a Marshall classification score of IV and a Rotterdam classification of 2. The CT scan showed an open comminute fronto‐parietotemporal left fracture and a fronto‐parietotemporal subdural hematoma that was 2 cm in diameter; in addition, the CT scan showed a fronto‐basal right contusion, a temporal contusion, and a small right temporal epidural hematoma. He also presented with multiple facial fractures that were addressed after the neurological situation was resolved. He underwent a bilateral fronto‐temporoparietal decompressive craniectomy 3 hours after the accident (Figure 3). We did not place a ventricular drain in this case. We started on the left side to resolve the transtentorial herniation. The mydriasis was reversed after the decompressive craniectomy was performed on the left side. We also evacuated the extracerebral hematomas on both sides. The patient was kept under sedation and paralysis and achieved an intracranial pressure of 8 mm Hg. He also presented with pneumonia (Figure 2) that required ventilatory assistance. The patient underwent a tracheostomy. After 4 weeks in the intensive care unit, the patient was admitted to the neurosurgical floor, where he received physical therapy. A cranioplasty repair was performed 9 weeks after the accident. An autologous graft was used on the right side. On the left side, a PEEK customized cranial implant was used. Six months later, the patient presented with an extended Glasgow Outcome Scale score of 8, indicating good upper recovery, and a Disability Rating Scale of 0.

## 3 RESULTS

Both patients underwent early bilateral fronto‐temporoparietal decompressive craniectomies with augmentative duraplasty and early cranioplasty repairs; after 6 months, both patients scored 8 on the extended Glasgow Outcome Scale, indicating good upper recovery, and had a modified Rankin Scale score of 0. The median intracranial pressure values of the two patients were 10 and 8 mm Hg. In the first case, the sizes of the extracranial cerebral herniations were 2.1 on the left side and 2.2 cm on the right side, with bone anteroposterior diameter openings of 13 and 12.5 cm, respectively. In the second case, the bone anteroposterior diameter openings were 12 cm on the left side and 11 cm on the right side, with cerebral herniations measuring 0.6 and 1.3 cm, respectively.

## 4 DISCUSSION

Ahmed et al described an early decompressive craniectomy as one that is performed within 4 hours of hospital admission, but craniectomies within the first 4 hours have not shown better outcomes than those performed within the first 24 hours^18^; in contrast, it has been shown that early craniectomies (less than 24 hours) could improve the long‐term outcomes of patients with refractory raised intracranial cerebral pressure and restore the blood flow after moderate or severe traumatic brain injuries^19^ (Table 1). In our cases, one patient was treated with a bilateral fronto‐temporoparietal decompressive craniectomy within the first 4 hours, and the other patient was treated within the first 24 hours; both patients presented the same outcomes, as shown in the study from Ahmed et al Zweckberger et al suggest that an early craniectomy may reduce secondary injuries to the brain.^20^

On the other hand, these patients were very young and had no comorbidities. It has been suggested that young patients could achieve good outcomes with standard decompressive craniectomies.^1^ This theory means that even if standard decompressive craniectomies were performed, the same outcomes as the ones we achieved could be obtained. Future randomized studies with young patients are necessary to show that bilateral fronto‐temporoparietal decompressive craniectomies are effective in achieving good outcomes.

Taking into account that the patients are young and that they underwent early decompressive craniectomies, the results were not surprising. In the rescue intracranial pressure trial, the mean intracranial pressure in the surgical group after randomization was 14.5 mm Hg (1.7‐18.0 mm Hg),^9^ and the same measure in the DECRA trial was 14.4 ± 6.8.^6^ The intracranial pressures of our patients were similar to these values; thus, bilateral fronto‐temporoparietal decompressive craniectomies probably do not play a role in achieving better control of intracranial pressure than other decompressive craniectomy techniques, but the patients did present good functional outcomes. Sauvigny et al^21^ investigated the course of ICP values with respect to neurological outcomes. They found in a cohort study that the ICP values were higher in the unfavorable outcome group than in the favorable outcome group. In the latter group, the ICPs remained below 15 mm Hg.^22^ In our patients, the ICPs achieved after decompressive craniectomy were approximately 10 and 8 mm Hg; therefore, this fact, in combination with the young age of patients, most likely played a role in achieving good functional outcomes.

Stiver et al suggested that decompressive craniectomies could lead to axonal stretching, especially because of herniations through the bone defects of the brain, chiefly in the cases where the decompressive craniectomy is small.^22^ This effect could be diminished by the augmentation of the area removed through craniectomy, as it can be accomplished by a bilateral fronto‐temporoparietal decompressive craniectomy.

Flint et al measured extracranial cerebral herniations as the diameter of brain extending beyond a straight line drawn between the outer table edges of the craniectomy defect. In their series of decompressive craniectomies, the mean diameter of the bone opening was 13.9 ± 1.2 cm, and the mean diameter of external cerebral herniation was 2.1 ± 0.9 cm.^23^ In the first case, the bone openings were 13 and 12.5 cm with herniations of 2.1 and 2.2 cm, respectively. In the second case, the bone openings were 12 and 11 cm with herniations of 0.6 and 1.3 cm, respectively. These values are smaller than those obtained with conventional craniectomy, but it would be necessary to prove it with a long series of cases.

Moreover, it is known that a potential adverse effect is the compression of the cortical veins within the herniated segment of the brain and the subsequent venous infarction of the herniated tissue. Increasing the craniectomy area with a bilateral fronto‐temporoparietal decompressive craniectomy can also reduce this effect. The patients in this study did not present with venous infarctions of the herniated brain. An augmentative duraplasty with DuraGen® was also performed, and the patch was placed between the arachnoid membrane and the opened dura mater. It may be that this patch layer partially prevents the compression of the veins with the dura mater edge.

The margin of decompression has a positive correlation with the bone flap diameter and the bone window area,^24^ and it has a significant relationship with mortality. There is an increase in mortality in patients with decompressive craniectomies that measure less than 10 cm in terms of the anteroposterior diameter.^25^ By performing a bilateral fronto‐temporoparietal decompressive craniectomy, it is possible to achieve a large craniectomy area, which could lead to a reduction in mortality; however, the optimal size of the craniectomy that can balance an optimal amount of decompression without increasing the risk of complications is still unknown. The bilateral approach, such as the bifrontal decompressive craniectomy, may have more complications.^9^ This makes it necessary to take the risk of infection into account. The use of bilateral approaches could hypothetically increase the risk of infection. Additionally, in both cases, an augmentative duraplasty was performed using a dura substitute with foreign synthetic material, which is associated with an increased risk of infection.^26^ In both cases, the superficial temporal artery was preserved. Preserving this vascular structure decreases the risk of wound infection. The patients did not present with wound infections, but the results regarding this issue cannot be taken into account, as our findings do not provide statistical significance.

In addition, the Marshall scale shows that there is a direct relationship between these four diagnostic categories and the mortality rate.^27^ The patients that presented with Marshall scores of IV would be predicted to have poor outcomes, but it is suggested that this scale could overestimate the number of poor outcomes from traumatic brain injury patients.^28^ It is also known that patients with Rotterdam scores of 5 or 6 had an 80% chance of experiencing expansions of their hemorrhagic contusions following a decompressive craniectomy.^23^ The patients presented in this manuscript scored 3 and 2 in the Rotterdam Scale; therefore, based on these scores, it was not possible to predict whether this technique could avoid the hemorrhagic progression of contusions.

The optimal timing of a cranioplasty after the decompressive craniectomy has not been well established, but it has been shown that earlier cranioplasty is associated with fewer extra‐axial fluids.^29^ Hygromas are generally ipsilateral to the skull defect with volumes ranging from 10 to 120 mL. One of the hypotheses for the mechanism of hygromas is the altered cerebrospinal fluid dynamics,^30^ but some authors have suggested that the increased cerebral perfusion pressure that accompanies a decompressive craniectomy may play a role.^31^ Bilateral fronto‐temporoparietal decompressive craniectomies could increase this effect on the perfusion pressure as it creates more space; nevertheless, the patients did not present with any hygromas.

The patients underwent cranioplasty repairs within the first 3 months after their decompressive craniectomy was performed, with the objective to not only avoid the extra‐axial fluid collections, taking into account the hypothesis of altered cerebrospinal fluid dynamics, but also avoid the motor trephine syndrome deficits that could appear 5 months after the decompressive craniectomy.^22^

A fronto‐temporoparietal decompressive craniectomy may relieve the tamponade effect on the contralateral bleeding site. 14, 15 A bilateral fronto‐temporoparietal decompressive craniectomy could avoid effusion of the contralateral extracerebral hematomas. The negative relationship between elevated intracranial pressures and unfavorable outcomes has been recognized in the past 20 years and was recently well demonstrated. However, the rescue intracranial pressure trial showed that decompressive craniectomies reduce mortality, but patients present high degrees of disability.^8^ The patients in this study had complete recoveries with GCS‐E scores of 8, indicating good upper recovery. We believe that this is due to the reduction in the diameter of herniation of the brain tissue, which reduces neuronal stretching.

### 4.1 Limitations

We acknowledge the limitations of this study. This is not an outcome study. This is a technical note based on two cases, so it cannot provide statistical significance for the data; however, it can give an explanation about why conventional craniectomies do not provide good functional outcomes and could lead to further investigations.

## 5 CONCLUSIONS

The augmentation of contralateral mass lesions from fronto‐temporoparietal decompressive craniectomies is well known. A bilateral fronto‐temporoparietal decompressive craniectomy could avoid this effect, and it should be considered at least in patients with diffuse injuries that present with contralateral extracerebral hematomas that could effuse in young patients.

This kind of decompressive craniectomy provides different directions for the brain to expand so that the level of brain tissue herniation through the craniectomy is low. This probably reduces neuronal stretching, which could play a role in patient disability outcomes, but decompressive craniectomies do not achieve better control of intracranial pressure compared to other kinds of decompressive craniectomies.

An augmentative duraplasty with nonautologous grafts positioned in between the opened dura and arachnoid, as well as an early cranioplasty, also plays a role in achieving good outcomes; preserving the superficial temporal artery can also help avoid wound infections.

A bilateral fronto‐temporoparietal decompressive craniectomy can provide a large craniectomy area that could allow for good functional outcomes with severe traumatic brain injuries; however, further studies are necessary to address the rate of complications.

## HIGHLIGHTS

Bilateral fronto‐temporoparietal decompressive craniectomies are not related to good control of intracranial pressure.

Bilateral fronto‐temporoparietal decompressive craniectomies provide a large craniectomy area that decreases the amount of brain tissue that herniates through the skull, leading to a decrease in the neuronal stretching effect, which is probably related to good functional outcomes.

## CONFLICT OF INTEREST

The authors declare that they have no financial or other conflicts of interest in relation to this research and its publication.

## AUTHORS’ CONTRIBUTION

RP‐A and KS‐D: conceived of the presented idea, contributed in the decision‐making process, and performed surgeries. RP‐A: took the lead in writing the manuscript. Both authors provided critical feedback and helped shape the manuscript.
